# *Bacopa monnieri* Extract (CDRI-08) Modulates the NMDA Receptor Subunits and nNOS-Apoptosis Axis in Cerebellum of Hepatic Encephalopathy Rats

**DOI:** 10.1155/2015/535013

**Published:** 2015-08-27

**Authors:** Papia Mondal, Surendra Kumar Trigun

**Affiliations:** Biochemistry Section, Department of Zoology, Banaras Hindu University, Varanasi 221005, India

## Abstract

Hepatic encephalopathy (HE), characterized by impaired cerebellar functions during chronic liver failure (CLF), involves N-methyl-D-aspartate receptor (NMDAR) overactivation in the brain cells.* Bacopa monnieri* (BM) extract is a known neuroprotectant. The present paper evaluates whether BM extract is able to modulate the two NMDAR subunits (NR2A and NR2B) and its downstream mediators in cerebellum of rats with chronic liver failure (CLF), induced by administration of 50 mg/kg bw thioacetamide (TAA) i.p. for 14 days, and in the TAA group rats orally treated with 200 mg/kg bw BM extract from days 8 to 14. NR2A is known to impart neuroprotection and that of NR2B induces neuronal death during NMDAR activation. Neuronal nitric oxide synthase- (nNOS-) apoptosis pathway is known to mediate NMDAR led excitotoxicity. The level of NR2A was found to be significantly reduced with a concomitant increase of NR2B in cerebellum of the CLF rats. This was consistent with significantly enhanced nNOS expression, nitric oxide level, and reduced Bcl2/Bax ratio. Moreover, treatment with BM extract reversed the NR2A/NR2B ratio and also normalized the levels of nNOS-apoptotic factors in cerebellum of those rats. The findings suggest modulation of NR2A and NR2B expression by BM extract to prevent neurochemical alterations associated with HE.

## 1. Introduction

The patients with liver cirrhosis develop a serious nervous system disorder known as hepatic encephalopathy (HE) [1]. It is characterized by wide spectrum of neuropsychiatric symptoms related to motor dysfunction, cognitive impairment, and disturbed sleep wake cycle [2–4]. Most of the liver cirrhotic patients have been found to show minimal to overt HE symptoms [5, 6], characterized mainly by the impaired motor functions [2, 7–9], which is considered to be associated with deranged cerebellar functions [10, 11]. Some recent findings from our lab also suggest that cerebellum shows greater susceptibility to undergo neurochemical changes in the models of chronic type HE [12–14]. Therefore, cerebellum was selected for the present study.

Based on the studies conducted to understand pathophysiology of HE, it has been suggested that the level of glutamate, an important excitatory neurotransmitter, increases in the synaptic cleft due to the increased blood and brain ammonia level during liver dysfunction, resulting into overactivation of the ionotropic N-methyl-D-aspartate receptors (NMDAR) [1]. And there is a general agreement that activation of glutamate-NMDA receptor-nNOS pathway constitutes main neurochemical aberrations associated with HE [1, 14].

Functional NMDAR, a tetrameric protein complex, comprises of two subunits of a constitutive glycine binding NR1 and remaining two of glutamate binding NR2 from amongst NR2A, NR2B, NR2C, and NR2D subunits. Importantly, combination of different NR2 subunits is suggested to confer unique electrophysiological properties to this neurotransmitter receptor [15, 16]. For example, alterations in the ratio of NR2A versus NR2B of NMDAR have been found to be associated with the changes in long term potentiation (LTP) and long term depression (LDP) functions during hippocampal plasticity involved in memory consolidation [17]. In addition, NR2A dominating combination of NMDAR is demonstrated to provide neuroprotection but NR2B rich NMDAR is known to drive the postsynaptic neuron towards apoptosis during glutamate excitotoxicity [14]. Thus, regulating NMDAR function by altering its composition, without pharmacological blockage of the channel, could be a unique and novel cerebral mechanism to prevent NMDAR overactivation led neurological disorders.

It is now evident that neuronal nitric oxide synthase (nNOS), via modulating NO level in the brain cells, plays critical roles in transmitting NMDAR led neurophysiological changes under different pathophysiological conditions [18, 19]. A threshold level of NO is essential to activate NO-cGMP signaling to maintain NMDAR dependent memory consolidation and cognition functions [18]. However, excess of NO is known to induce apoptosis and neuronal death [20, 21]. Such multimodal roles of NMDAR-nNOS axis in brain cells are orchestrated by a molecular link between NMDAR and nNOS protein. The NR2 subunits of NMDAR complex, through their tSXV motifs, connect with the postsynaptic density protein-95 (PSD-95), which in turn, via its PDZ domain, interacts with the nNOS in the postsynaptic neurons [19, 22]. This constitutes the main mechanism of NMDAR activity led NO production and subsequent changes in the neuronal functions. Particularly during HE, activation of NMDAR leads to the increased calcium influx which in turn activates nNOS and thereby overproduces NO in the postsynaptic neurons [1, 9, 13]. Thus increased glutamate led NMDAR activation, via activation of nNOS, is considered critically involved in developing HE associated neuropsychiatric problems in the patients/animals [13, 23, 24].

Obviously NMDAR becomes choice of an important therapeutic target for the neurobiologists for HE management [25, 26]. Some earlier studies conducted* in vivo* and* in vitro* using NMDAR antagonists indeed demonstrated desirable results; however, this approach was found to produce undesirable neurological complications during the clinical trials [25, 27]. This is not surprising as NMDAR activity is critical for maintaining normal neurophysiology including higher order brain functions and memory consolidation mechanisms [17]. Therefore, instead of blocking NMDAR channel, modulation of NMDAR activity by alterations in its functional composition and downstream signaling seems to be of special scientific merit. However, this evolving concept needs to be examined in the animal models with excitotoxic neurological problems. Since development of HE is related with NMDAR led excitotoxicity [1] and that herbal formulations are now evident to modulate brain chemistry in many ways, the present work was undertaken to evaluate whether* Bacopa monnieri *(BM) extract, a known neuroprotectant, is able to modulate NMDAR composition and related downstream events in cerebellum of the CLF induced HE rats.

Amongst a good number of herbal drugs available,* Bacopa monnieri *extract has been widely evaluated as a memory enhancer, adaptogenic, anti-inflammatory, analgesic, antipyretic, sedative, and antiepileptic agent [28]. Also, some studies suggest its neuroprotective roles against epilepsy (neuroexcitotoxic outcome) by modulating serotonergic receptor [29], and against Parkinson's and Alzheimer's disease via altering dopaminergic signaling [30] and cholinergic [31] receptors, respectively. Moreover, molecular mechanisms underlying these BM extract effects remain largely unexplored.

Importantly, though information is limited, efficacy of BM extract has also been shown against glutamate toxicity via modulating NMDAR1 gene expression and in turn affecting glutamatergic signaling [32]. In our previous reports, we have observed a direct association between overexpression of the constitutive NR1, nNOS activation, and enhanced NO production in cerebellum of the CLF rats exhibiting HE characteristics [2, 13, 14]. Importantly, we could also observe reciprocal expression of NR2A and NR2B in the cerebellum of those rats (data from this paper). This tempted us to investigate whether administration of BME is able to alter this unusual NR2A/2B composition and thus NMDAR-nNOS pathway in the cerebellum of the HE rats.

## 2. Materials and Methods

### 2.1. Chemicals

Chemicals used were of analytical grade supplied by E-Merck and Sisco Research Laboratory (India). Acrylamide,* N,N*′-methylenebisacrylamide, N,N,N′N′-tetramethylethylenediamine (TEMED), phenyl methyl sulphonyl fluoride (PMSF), bromophenol blue, and Ponceau were purchased from Sigma-Aldrich, USA. Primary antibodies used were procured from the following companies: rabbit monoclonal *β*-actin from Sigma Aldrich, rabbit monoclonal anti-NR2B from Invitrogen, rabbit monoclonal NR2A from Epitomics, rabbit polyclonal Bcl2 and Bax from Cell Signaling Technology, and rabbit polyclonal nNOS from Santa Cruz Biotechnology. Rabbit and mouse horseradish peroxidase (HRP) conjugated secondary antibodies were obtained from Genei. ECL western blotting detection kit was purchased from Thermo Scientific.

The ethanolic extract of* Bacopa monnieri* extract (BM/CDRI-08), containing 64.28% bacoside A and 27.11% bacoside B, was obtained from the Lumen Research Foundation, Chennai, India.

### 2.2. Animals

Inbred adult female albino rats weighing 150–160 g were used in this study. The rats were kept in separate cages, fed with the recommended diet, and maintained at standard conditions of 12 h light and dark period at room temperature (25 ± 2°C) in an animal house. The use of animals for the present study was approved by the Institutional Animal Care and Use Committee (IACUC); Animal Ethical Committee (AEC) of the Banaras Hindu University, Varanasi.

### 2.3. Induction of Chronic Liver Failure (CLF)/HE and Treatment Schedule

The CLF/HE model of neuroexcitotoxicity in adult albino rats was induced by the administration of thioacetamide (TAA) as standardized previously [2]. For this, rats were randomly divided into three groups with 6 rats in each. Group A: control (C), administered with 0.9% saline i.p, once daily for 14 days; Group B: CLF/HE group, administered with 50 mg/Kg bw TAA i.p once daily for 14 days; Group C: CLF + Bacopa extract (CLF + BM). Rats in Group C were orally administered with ethanolic extract of BM extract (CDRI-08; 200 mg/Kg bw), suspended in 1% gum acacia, once daily starting from 8th day onwards till 14th day, and were administered 4 h after the TAA treatment. The dose of BM was selected which was able to recover TAA induced neurobehavioural deficit in the rats. All the rats were sacrificed on 15th day. The cerebellum was dissected out and stored at −80°C for further experiments.

### 2.4. Preparation of Cerebellar Extracts

As described earlier [13], mitochondria free cerebellar extracts were prepared in an extraction medium consisting of 400 mM sucrose, 1 mM EDTA, 0.2 mM benzamidine, 0.1 mM phenylmethylsulfonyl fluoride (PMSF), and 0.02% heparin. The extracts were centrifuged initially at 12000 ×g for 15 min and finally at 19000 ×g for 45 min at 4°C. The supernatant obtained was collected as the cytosolic fractions. The protein content in the tissue extract was measured by Lowry method [33] using BSA as standard.

### 2.5. Western Blotting

As described previously [13], the cytosolic fractions containing 60 *μ*g protein/lane were separated on 10% SDS-PAGE and electrotransferred to nitrocellulose membrane at 50 mA and run overnight at 4°C. Protein transfer was checked via Ponceau staining. The membrane was then placed in a blocking solution of 5% skimmed milk in 1X PBS for 2 h followed by washing in PBS 3 times. The membranes were then separately processed for immunodetection of NR2A, NR2B, nNOS, Bcl2, and Bax using monoclonal/polyclonal anti-NR2A (1 : 1000), anti-NR2B (1 : 1000), anti-nNOS (1 : 500), anti-Bcl2 (1 : 1000), and anti-Bax (1 : 500), respectively. HRP conjugated secondary antibody was used for final detection of the proteins using ECL western blotting detection kit. *β*-actin, used as loading control, was detected using a monoclonal anti-*β*-actin peroxidase antibody. The bands were quantified and analyzed using gel densitometry software AlphaImager 2200. The photographs in the figure are representatives of the three western blot repeats.

### 2.6. Nitric Oxide (NO) Estimation

Nitric oxide level was measured by estimating total nitrite (NO_2_) and nitrate (NO_3_) content in the tissue extracts as described earlier [13] using the method of Sastry et al. [34]. Briefly, tissue fractions (100 *μ*L), NaNO_2_, and KNO_3_ standards (0.1 mM each) were mixed separately with 400 *μ*L of 50 mM carbonate buffer (pH 9). For NO_3_ estimation, activated copper-cadmium alloy (150 mg) was added and incubated for an hour at 37°C. The reaction was stopped using 100 *μ*L each of 0.35 M NaOH and 120 mM zinc sulphate. After centrifugation, 400 *μ*L supernatant was incubated with the Griess reagent: 200 *μ*L 1% sulphanilamide prepared in 2.5% H_3_PO_4_ and 200 *μ*L 0.1% N-(1-naphthyl)-ethylenediamine. Absorbance was recorded at 545 nm. For NO_2_ estimation, similar procedure was followed except addition of the copper-cadmium alloy.

### 2.7. Statistical Analysis

The data have been expressed as mean ± SD. For two group comparisons, statistical analysis was performed using unpaired Student's *t* test. A probability of *P* < 0.05 was taken as a significant difference between the groups. Each of the experiments was repeated thrice.

## 3. Results

### 3.1. Effect of BM Extract on NR2A and NR2B Expression

**Figure 1:**
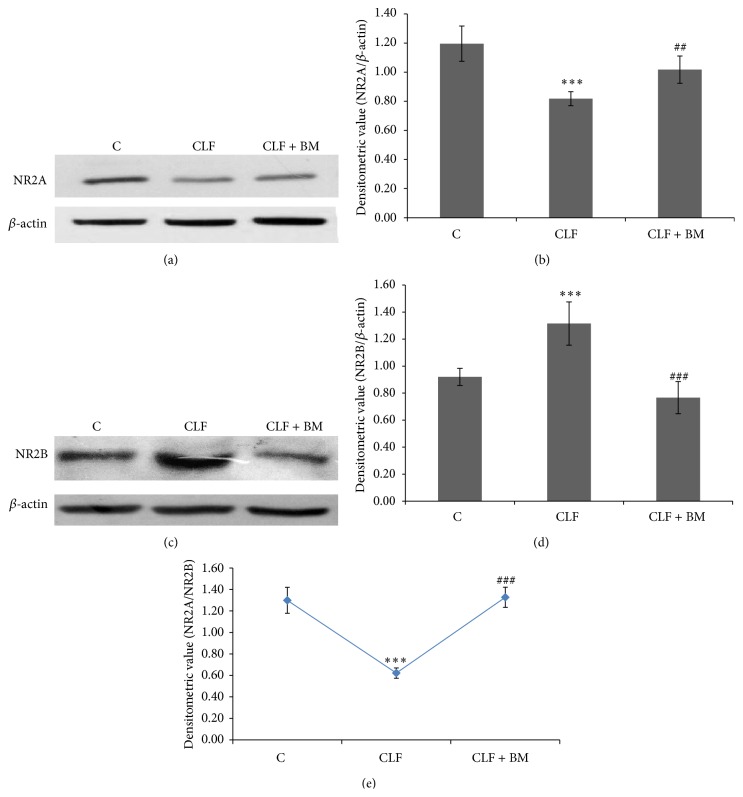
Expression profile of NR2A ((a) and (b)) and NR2B ((c) and (d)) in cerebellum of the CLF and BM extract treated CLF rats. Western blot analysis was performed as described in the method text. The level of *β*-actin was probed as the loading control. In panels (b), (d), and (e), normalized densitometric values of NR2A/*β*-actin, NR2B/*β*-actin, and NR2A/NR2B have been presented as mean ± SD from three western blot repeats. ^***^ *P* < 0.001; (control versus CLF rats) ^##^ *P* < 0.01, ^###^ *P* < 0.001; (CLF versus CLF + BM rats). C: control, CLF: chronic liver failure, CLF + BM: chronic liver failure +* Bacopa monnieri* extract.

The combination of constituent NMDAR subunits is evident to impart unique neurophysiological role of this glutamate receptor. The NR2A dominating combination is known to mediate neuroprotection whereas those of NR2B induce neuronal death. According to Figures 1(a) and 1(b), as compared to the control rats, level of NR2A is found to be significantly reduced (*P* < 0.001) with a concomitant increase in NR2B level in the cerebellum of the CLF rats (Figures 1(c) and 1(d)), resulting in a significant decline in NR2A/2B ratio (Figure 1(e)). However, this pattern is observed to be recovered back with a significant enhancement of NR2A (Figures 1(a) and 1(b)) and a decline of NR2B (Figures 1(c) and 1(d)), resulting into attaining a control level NR2A/2B ratio (Figure 1(e)) in the cerebellum of the CLF rats treated with the BM extract.

### 3.2. Effect of BM Extract on nNOS and Nitric Oxide (NO) Production

**Figure 2:**
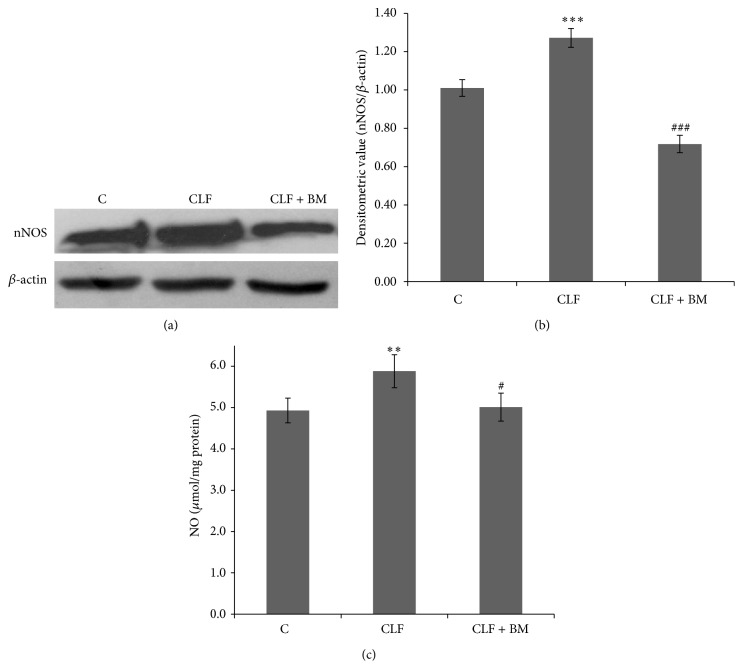
Expression profile of nNOS ((a) and (b)) and NO level (c) in cerebellum of the CLF and BM extract treated CLF rats. Western blot analysis of nNOS was performed as described in the text of methods. In panel (b), normalized densitometric values of nNOS/*β*-actin have been presented as mean ± SD from three western blot repeats. ^**^ *P* < 0.01, ^***^ *P* < 0.001; (control versus CLF rats) ^#^ *P* < 0.05, ^###^ *P* < 0.001; (CLF versus CLF + BM rats). C: control, CLF: chronic liver failure, CLF + BM: chronic liver failure +* Bacopa monnieri* extract.

Neuronal NOS (nNOS) has a direct molecular link with NMDAR and therefore, it is considered to be the main determinant of NMDAR activation based downstream signaling in the postsynaptic neurons. Accordingly, overactivation of nNOS is considered associated with the neuronal changes associated with NMDAR led excitotoxicity. As depicted in Figures 2(a) and 2(b), nNOS expression is observed to be enhanced significantly (*P* < 0.001) in the cerebellum of the CLF rats as compared to the control group rats. Moreover, due to the oral administration of BM extract, level of this enzyme is observed to be reduced up to the control value (Figures 2(a) and 2(b)). Such a pattern could coincide well with the similar changes in the NO level in cerebellum of the CLF and BM extract treated CLF rats (Figure 2(c)).

### 3.3. Effect of BM Extract on the Expression of Bcl2 and Bax

**Figure 3:**
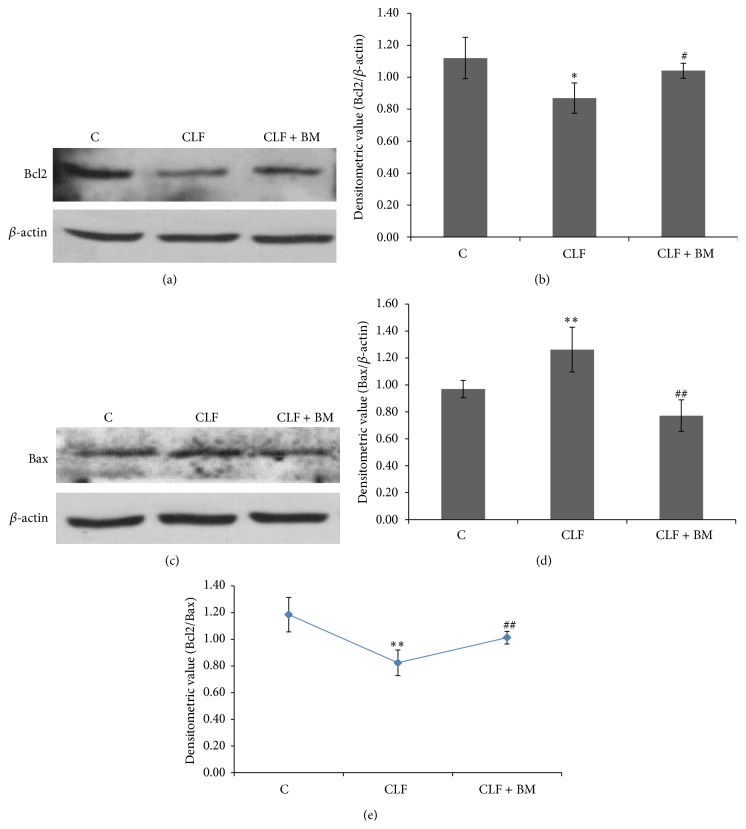
Expression profile of Bcl2 ((a) and (b)) and Bax ((c) and (d)) in cerebellum of the CLF and BM extract treated CLF rats. Western blot analysis of Bcl2 and Bax was performed as described in the text of methods. The level of *β*-actin was probed as the loading control. In panels (b), (d), and (e), normalized densitometric values of Bcl2/*β*-actin, Bax/*β*-actin, and Bcl2/Bax ratio, respectively, have been presented as mean ± SD from three western blot repeats. ^*^ *P* < 0.05, ^**^ *P* < 0.01; (control versus CLF rats) ^#^ *P* < 0.05, ^##^ *P* < 0.01; (CLF versus CLF + BM rats). C: control, CLF: chronic liver failure, CLF + BM: chronic liver failure +* Bacopa monnieri* extract.

Bcl2 (antiapoptotic) and Bax (proapoptotic) ratio serves as a rheostat to determine cell susceptibility to apoptosis. According to Figures 3(a) and 3(c), as compared to the control group rats, level of Bcl2 is found to be declined significantly (*P* < 0.05) with a concomitant increase in the Bax level resulting in a significant decline (*P* < 0.01) in Bcl2/Bax ratio (Figure 3(e)) in cerebellum of the CLF rats. However, after treatment with BM extract, the pattern of Bcl2/Bax ratio is observed to regain its normal range in cerebellum of those CLF rats (Figure 3(e)).

## 4. Discussion

NMDA receptor overactivation is considered a common neurochemical event associated with a number of brain dysfunctions like epilepsy, ischemia, drug abuse, and HE [23, 26, 35–37]. However, NMDAR blockage is evident to be a poor therapeutic target for managing such excitotoxic conditions [25, 27]. In this background, while understanding neurochemical basis of CLF induced HE, we observed a clear shift in the expression of glutamate binding NMDAR subunit from a NR2A dominating combination to a NR2B rich combination in cerebellum of the CLF rats (Figures 1(a) and 1(c)). It is now becoming clearer that NR2A rich NMDAR imparts neuroprotection and that of NR2B dominating combination initiates neuronal damage and apoptosis [16, 38–40]. This is because NR2B has been reported to show delay in gating kinetics in comparison to that of NR1/NR2A combination resulting in increased Ca^2+^ influx and thus rapid activation of the downstream signaling [41]. In the present context, a significant decline in Bcl2 with a concomitant increase in Bax level in cerebellum of the CLF rats (Figure 3) also hints for a direct association between NR2B dominating NMDAR composition and altered neurochemistry of cerebellum of the CLF rats. Furthermore, alterations in oxidative and nitrosative factors in the postsynaptic neurons are considered to be the main downstream mediators of such unusual NMDAR activations [1] and it has been reported that cerebellum shows greater susceptibility to undergo oxidative and nitrosative stress during HA and CLF led excitotoxicity [12, 13].

It has been described that lesion in cerebellum impairs acquisition of memory consolidation and deranges motor functions [10, 11]. The CLF rats used in this experiment have recently been reported to show cognitive impairment and deficit in motor functions as well [2]. Since such neurochemical alterations leading to neurobehavioural changes are known to emanate from abnormal NMDAR activity [1], it is argued that a shift from a NR2A combination to a NR2B combination of NMDAR in the cerebellum of the CLF rats might be accountable for developing HE associated symptoms observed in these CLF rats [2]. Moreover, keeping aside these explanations, the findings provided a basis to alter NMDAR constitution, instead of shutting this ion-channel off, as a therapeutic option to bring recovery from the HE symptoms.

To test this hypothesis, BM extract was chosen due to the two reasons. Firstly, amidst scarcity of the safer neuropharmacological agents, this plant extract is demonstrated to improve neuronal functions by modulating brain chemistry in many ways [28]. Secondly, BM is now evident to modulate activity of the neurotransmitter receptors like serotonergic receptors during epilepsy [29] and dopaminergic and cholinergic signaling in Parkinson's and Alzheimer's diseases [30, 31]. However, report is limited on modulation of NMDA receptor activity by BM. We observed a remarkable shift from a declined ratio of NR2A/NR2B (neurodegeneration supportive combination) in the cerebellum of CLF rats towards their normal level when these CLF rats were administered with BM extract (Figure 1(e)). This finding suggested that BM is able to alter constitution of the functional NMDAR tetramer by differential expression of the two glutamate binding subunits in cerebellum of the CLF rats. Indeed, in an epilepsy model, it has been demonstrated that BM could alter NR1 gene expression [32], thus suggesting BM as a modulator of NMDAR subunit expression. A similar finding on modulation of NR1 by BM was reported in a hypoxia model also [42]. Importantly, such a change in NR1 expression was found accountable for modulating glutamatergic signaling in cerebellum of those rats [32]. Thus, it is argued that shifting from a neurodegenerative NR2B overexpression towards the neuroprotective NR2A level due to the treatment with BME (Figure 1) could also account for preventing downstream undesirable neurochemical changes in cerebellum of the CLF rats.

In a number of excitotoxic models, including CLF led HE, nNOS activation is considered as the most common event after NMDAR activation [13, 43]. It is initiated by influx of Ca^2+^ which overactivates this enzyme to produce excess NO in the postsynaptic neurons [44]. NO is a molecule of pleiotropic effects; however, when produced in excess in the brain cells, it uses more than one mechanism to induce neuronal dysfunction [20, 21]. A milder increase in NO level is likely to initiate mitochondrial dysfunction led neuronal apoptosis. Ratio of Bcl2 versus Bax is considered as the most effective regulators of mitochondrial dysfunction led apoptosis [45] and hence relative levels of both these proteins are considered as a reliable tool to assay whether a cell is likely to undergo internal apoptotic process [46]. In cerebellum of the CLF rats, a significantly increased level of nNOS coincides well with a similar increment in NO level (Figure 2). This is consistent with a significant decline in the Bcl2/Bax ratio (Figure 3). Moreover, all these factors were found to be reversed to regain their normal levels in the cerebellum when those CLF rats were treated with BM extract (Figures 1–3). Recent studies have also shown that BM extract downregulates Bax and upregulates Bcl2 and thus provides neuroprotection in several neurological disease models [47]. Thus, the findings of Figures 1–3 together advocate for concordant modulation of NMDA receptor constitution and nNOS led apoptotic activation by ethanolic extract of BM (CDRI-08) in cerebellum of the CLF rats.

## 5. Conclusion

Opposing roles of the two main glutamate binding subunits, NR2A and NR2B, of NMDA receptor advocate for modulation of NMDAR constitution, as an effective mechanism to normalize its excitotoxic effects without blocking its ion-channel activity. During recent past, studies on efficacy of* Bacopa monnieri* extract have been shown to prevent neurodegenerative diseases by modulating neurochemistry of the brain cells, but with little information about modulation of NMDAR overactivation led excitotoxicity. The present findings demonstrate that BM extract is able to modulate NMDA receptor signaling by bringing reciprocal changes in the expression of its two glutamate binding subunits, NR2A and NR2B, and thus provide a novel approach to normalize NMDAR overactivation effects without blocking this ion channel.
